# Efficacy of peptide receptor radionuclide therapy in patients with oligodiscordant gastroenteropancreatic neuroendocrine tumours

**DOI:** 10.1007/s00259-026-08042-7

**Published:** 2026-07-06

**Authors:** Claire Mok, Wallace Chow, Zahra Sabahi, Elizabeth J. Bernard, Paul J. Roach, Dale L. Bailey, Connie I. Diakos, Nick Pavlakis, David L. Chan

**Affiliations:** 1 Department of Medical Oncology, Royal North Shore Hospital, Sydney, NSW Australia; 2 Faculty of Medicine and Health, University of Sydney, Sydney, NSW Australia; 3 Department of Nuclear Medicine, Royal North Shore Hospital, Sydney, NSW Australia

**Keywords:** Neuroendocrine tumours, FDG PET, DOTATATE PET, Peptide receptor radionuclide therapy, Discordant disease, Overall survival, Progression free survival, FDG avid disease

## Abstract

**Background:**

The use of peptide receptor radionuclide therapy (PRRT) is well established in the treatment of advanced or unresectable neuroendocrine tumours (NETs) after progression on somatostatin analogues (SSA), with randomised clinical trials such as NETTER-1 and NETTER-2 showing improvement in progression free survival (PFS) as well as tumour response [12, 13]. Current landmark trials only considered patients suitable for PRRT if all known lesions displayed SSTR expression, via avidity on planar scintigraphy or Gallium-68 DOTATATE PET/CT. However, there is a select group of patients with oligodiscordant disease (3 or less lesions which are FDG avid but not DOTATATE avid) on dual DOTATATE/FDG PET imaging who may benefit from PRRT in combination with additional therapies such as liver directed therapy (LDT) or chemotherapy. There is currently no data regarding the efficacy and outcomes of PRRT in oligodiscordant disease. This study is the first to describe the treatment patterns and outcomes of patients with oligodiscordant disease receiving PRRT.

**Methods:**

A single-centre retrospective review was performed in patients with advanced gastroenteropancreatic NETs with oligodiscordant disease who received at least one cycle of PRRT with [177Lu]Lu–DOTA-TATE from 2020 to 2024. Safety was assessed by renal and haematological parameters (CTCAE v5.0) during PRRT, and at 3 months post completion of treatment. Response to treatment was evaluated on molecular imaging with Gallium-68 DOTATATE PET/CT. Kaplan-Meier method was used to perform median progression free survival (PFS), overall survival (OS), and time to next treatment (TTNT) analyses.

**Results:**

Thirteen patients met the inclusion criteria, the median age was 66 years, and 54% were male. Primary site: small bowel (7), pancreas (4), colorectal (2). WHO grade: none had grade 1 disease, ten grade 2 and three had grade 3 disease. Median OS was 27.3 months (95% CI 19.7 - not reached), median PFS 15.1 months (95% CI 7.1–20.1), and median TTNT 15.0 months (95% CI 6.7–39.6). One of 15 patients developed treatment related myeloid neoplasm on long term follow-up, confirmed by bone marrow biopsy. No patients developed significant renal toxicity on follow-up.

**Conclusion:**

In patients with advanced NETs with oligodiscordant disease identified on dual PET imaging, the combination of PRRT with systemic therapy or other therapies (surgery, liver directed therapy, radiotherapy) can be effective with acceptable toxicity and should be considered as a possible option for treatment in selected patients. The sequencing of treatment modalities and exact strategy employed is best decided at an experienced NET centre with multi-disciplinary input. Keywords: Neuroendocrine tumours, FDG PET, Dotatate PET, Peptide receptor radionuclide therapy, Discordant disease, Overall survival, Progression free survival, FDG avid disease

## Introduction

Neuroendocrine neoplasms (NENs) are an uncommon and heterogenous group of tumours which most commonly arise from the gastrointestinal tract, and pancreas. Gastroenteropancreatic NETs (GEPNETs) are categorised by the World Health Organisation histological grade, incorporating the mitotic count and Ki-67 index, which informs tumour aggressiveness and prognosis [1]. However, even in patients with the same grade, patients can have extremely varied clinical course [2], and additional prognostic biomarkers are required to guide optimal practice.

The combined interpretation of [68Ga]Ga-DOTATATE (DOTATATE) PET and [18F]fluoro-deoxyglucose (FDG) PET is a highly informative clinical tool in patients with metastatic GEPNETs [3–5]. Lower grade tumours often harbour strong somatostatin receptor (SSTR) expression and thus avidity on DOTATATE PET [6, 7]. They are less metabolically active, and can display less or no avidity on FDG PET. On the contrary, higher-grade tumours can harbour less SSTR expression and thus display less avidity on DOTATATE PET, whilst displaying more FDG avidity. This pattern of loss of DOTATATE avidity coupled with increased metabolic/FDG avidity (“discordant disease”) suggests the presence of de-differentiated, higher-grade pathology and predicts a poorer prognosis [8, 9]. Current literature validating the NETPET score based on dual PET imaging shows that DOTATATE positive/FDG negative disease portends the best prognosis (P1), concordant DOTATATE and FDG positive disease suggests an intermediate group (P2-4), and discordant DOTATATE negative/FDG positive disease (P5) suggests de-differentiated, higher grade disease and the worst overall survival [10, 11]. In particular, the presence of any volume of discordant disease portends a poorer prognosis [11]. Though not currently mandatory, obtaining dual PET scans is currently considered standard practice in many institutions [6], with more widespread adoption in the US/Canada mainly constrained by limited imaging availability.

Following histopathological diagnosis, and accurate staging with molecular imaging, advanced GEPNETs are most commonly treated with first-line somatostatin analogues (SSAs; octreotide or lanreotide), particularly in patients with high volume disease or carcinoid symptoms. After progression on first-line SSAs, the strategy for subsequent therapies is complex and based on grade, findings on cross-sectional and PET imaging, and patterns of progression. Currently, the armamentarium includes peptide receptor radionuclide therapy (PRRT), chemotherapy and oral targeted therapies (such as everolimus, sunitinib, and the more recently approved cabozantinib) [2, 22].

Amongst these options, PRRT is increasingly used in the treatment of advanced NETs, with 2 randomised phase III clinical trials (NETTER-1 and NETTER-2) establishing significant benefits in progression-free survival, both in the first and second-line setting [12, 13]. These two trials required patients to have avidity on either planar SSTR scintigraphy or DOTATATE PET on all tumour lesions, but did not mandate the use of FDG PET prior to PRRT. As administered PRRT will not accumulate in lesions which are not DOTATATE avid, current European Neuroendocrine Tumour Society (ENETs) and European Society for Medical Oncology (ESMO) guidelines recommend against use of PRRT in patients with discordant disease [14, 15]. In this cohort, systemic treatment such as oral targeted therapy (including everolimus, sunitinib, and cabozantinib) or chemotherapy is usually recommended. However, there may be a select group of patients whose disease is mostly DOTATATE avid, in whom the administration of PRRT may still provide significant clinical benefit.

In this paper, we hypothesize that patients with a small number of discordant lesions (i.e. those in whom most lesions remain DOTATATE avid) may still derive benefit from PRRT, potentially in combination with other treatments targeting these discordant lesions. The prefix “oligo-” in oncology has been used variably to describe anywhere from 1 to 5 lesions, such as in “oligoprogression” and “oligometastatic” disease [16, 17]. Given the lack of evidence of outcomes of patients with metastatic GEPNETs receiving PRRT, we took a more conservative approach and defined “oligodiscordant disease” as a PET phenotype where patients have at least 1, but no more than 3 discordant (FDG avid, DOTATATE non-avid) lesions on dual PET imaging. Currently, there is growing evidence supporting the efficacy of PRRT rechallenge in patients who have a good period of initial response (of at least 6 months) to PRRT. As such, patients with prior PRRT were also included to reflect real-world treatment patterns.

To our knowledge, the safety, efficacy and outcomes of PRRT, alone or in combination with other treatment modalities, in such “oligodiscordant” patients has not been described to date. Real world evidence is required to report the efficacy of PRRT in this “borderline” group. This evidence is increasingly more relevant given the potential for use of PRRT in higher grade tumours, which is associated with a higher incidence of discordant disease. As such, we aimed to report treatment patterns, safety, and outcomes of patients with progressive GEPNETs with oligodiscordant disease treated with PRRT at our centre.

## Materials and methods

### Patients and data collection

We conducted a retrospective study of all patients who were treated with at least 1 cycle of PRRT at a tertiary academic hospital in Sydney, Australia, between January 2020 and December 2024. Eligible patients had histologically confirmed advanced (unresectable or metastatic) GEPNET of any grade, with dual PET imaging identifying oligodiscordant disease, defined as 1–3 “discordant” lesions displaying wholly DOTATATE non-avid and wholly FDG avid disease. Patients were discussed at local multi-disciplinary tumour board (MTB), which included medical oncologists, interventional radiologists, nuclear medicine physicians, radiation oncologists, endocrinologists and surgeons.

Patient demographics, disease characteristics, number and sites of discordant disease, treatment administered (total cycles and dose of PRRT administered), treatment around the time of PRRT (defined as other treatments administered 4 weeks prior to, during, or within 3 months after PRRT), safety as assessed by haematological and renal parameters (CTCAE v5.0) during and post PRRT treatment were collected by chart review. Imaging data with baseline DOTATATE PET and FDG PET was reviewed by a senior nuclear medicine physician, and restaging imaging at approximately 3 months post PRRT (as is current standard of care) were collected. FDG PET scans were initially windowed at 0–7 (as is used in standard reporting for clinical practice), with dynamic windowing allowed in line with methods previously described in our 2017 publication [11].

The endpoints of the study were as follows: (1) Overall survival, defined as the time from cycle 1 day 1 (C1D1) of PRRT to date of death or last follow-up, (2) Progression-free survival, defined as time from C1D1 of PRRT to date of locally judged progression (progression defined as outcome of imaging review at MTB, and/or radiology and nuclear medicine reports), and (3) Time to next treatment (TTNT), defined as time from C1D1 of PRRT to date of commencement of subsequent anticancer treatment (systemic or local). If the event (OS, PFS or TTNT) was not reached, time to event was calculated to the last date of follow up. The date of data lock was October 9, 2025. Safety data was collected for Grade 3–4 haematological and renal toxicities including the diagnosis of treatment-related myeloid neoplasm and presented as a percentage of all included patients.

Descriptive statistics were used to describe demographic data, disease characteristics, toxicity and key imaging data. Survival data (OS, PFS and TTNT) was censored at last follow-up where relevant and estimated using the Kaplan-Meier method. The project was approved by the Northern Sydney Local Health District Human Research Ethics Committee (2024/ETH01540).

## Results

**Fig. 1:**
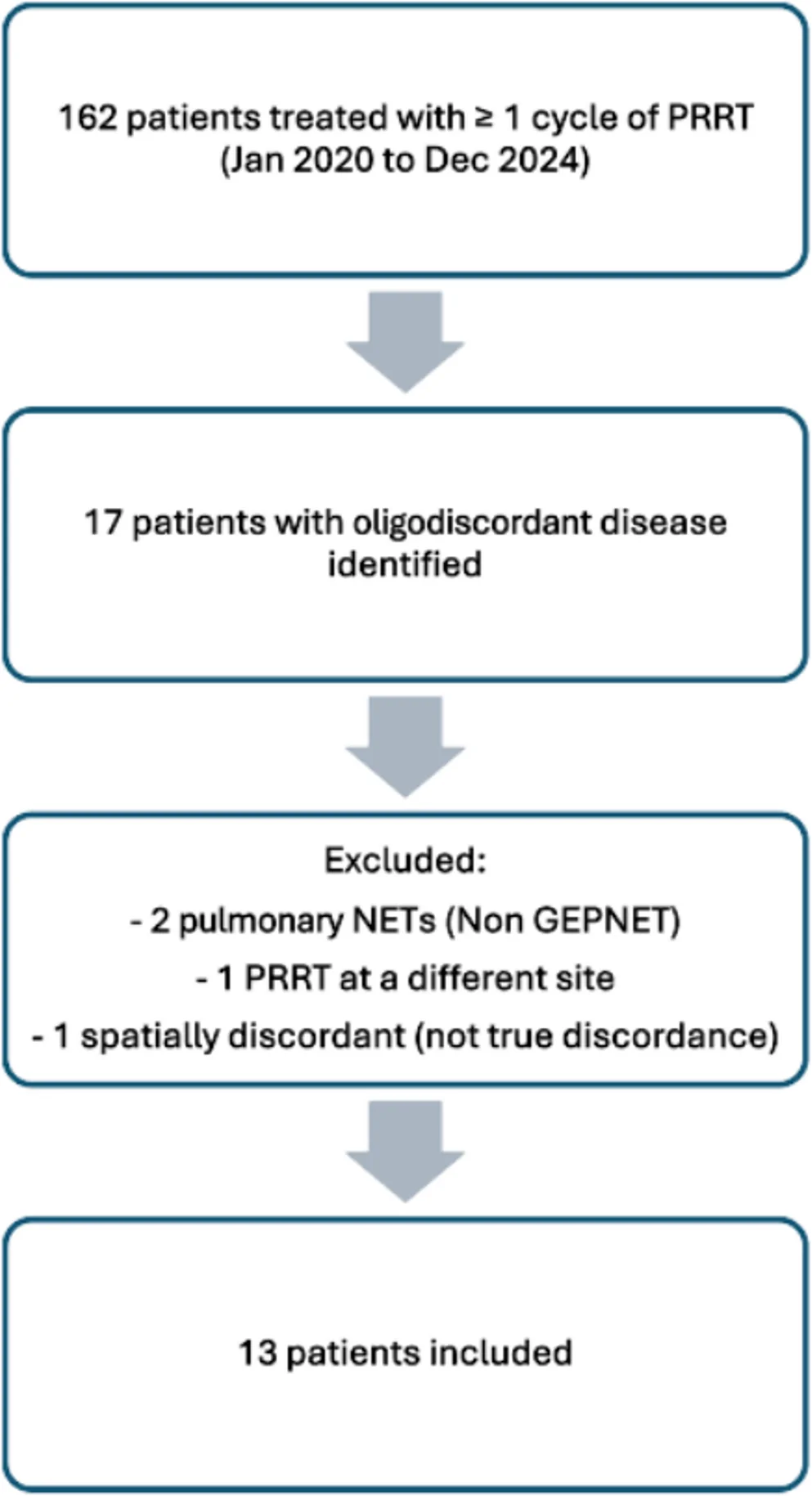
Patient flow chart for inclusion and exclusion criteria

Seventeen patients were identified with likely oligodiscordant disease on chart review, out of a total of 162 patients treated with at least one cycle of PRRT during the index time period. On further review of medical records, 2 patients were excluded for NET of non-GEP origin, and 1 patient was excluded as they had PRRT at a different site (Fig. 1). One patient had “spatially discordant” disease (that is, a metastatic lesion with some parts DOTATATE avid, and some parts FDG avid, DOTATATE non-avid) without other discordant sites and was excluded. A total of 13 patients were therefore eligible and included for detailed analysis.

### Demographics and clinicopathological characteristics

Baseline patient characteristics are detailed in Table 1. The median age of patients was 66 years, and 7 (54%) were male. The most common site of origin was the small bowel, followed by the pancreas. None of patients had grade 1 disease, 10 had grade 2, and 3 had grade 3 disease. The median Ki-67 index was 10% (range 3.5% − 58%). Included patients were heavily treated, with prior treatments including SSA (92%), LDT (69%), surgery (46%), prior PRRT (38%) and other treatments (8%) (Table 2).

Five patients had received prior PRRT, with 1 patient having had 2 courses of PRRT prior (to a total of 8 cycles of treatment). In the patients who had prior PRRT, no discordant disease was noted on workup for first or second course of PRRT. Eleven of the 13 patients had only one discordant lesion and the remaining 2 had two discordant lesions (Table 1).

**Table 1:** Baseline characteristics of patients

Baseline characteristics	*N* = 13
Age (median; range)	66 (47–78)
*Sex*	
Female Male	6 7
*Primary site*	
Small bowel Pancreas Colorectal	7 4 2
*Grade*	
1 2 3	0 10 3
Median Ki-67 index, (range)	10% (3.5–58)
*Number of oligodiscordant lesions*	
1 2 3	11 2 0
*Previous treatment*	
Surgery SSA Chemotherapy LDT (including SIRT, TACE, MWA) PRRT Other	6 (46%) 12 (92%) 6 (46%) 9 (69%) 5 (38%) 1 (8%)
*Stage at time of PRRT therapy*	
Locally advanced Metastatic	0 13

SSA – somatostatin analogues; LDT – liver directed therapy; SIRT – selective internal radiation therapy; TACE – trans-arterial chemo-embolization; MWA – microwave ablation

### Treatment trends and toxicity

PRRT and treatment characteristics are described in Table 2. Of the planned 4 cycles of PRRT, 8 of the 13 patients received the full course, 3 received 3 cycles and 2 received 2 cycles. Reasons for early cessation of treatment were: progressive disease in 2 patients, concomitant illness - new diagnosis of brain malignancy (1), and toxicity – fatigue and thrombocytopaenia (2).

**Fig. 2:**
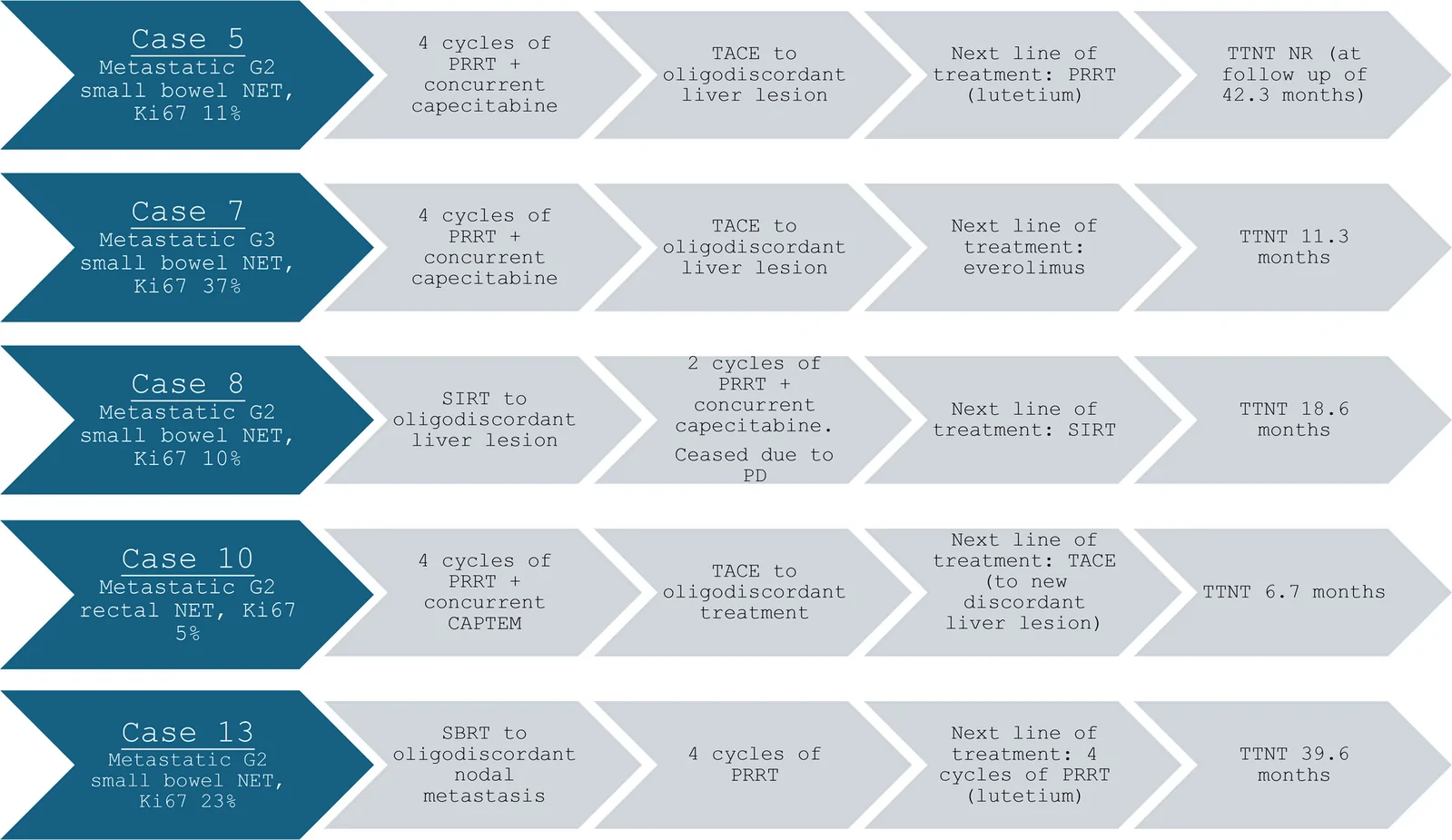
Peri-PRRT treatment sequencing of select patients

**Fig. 3:**
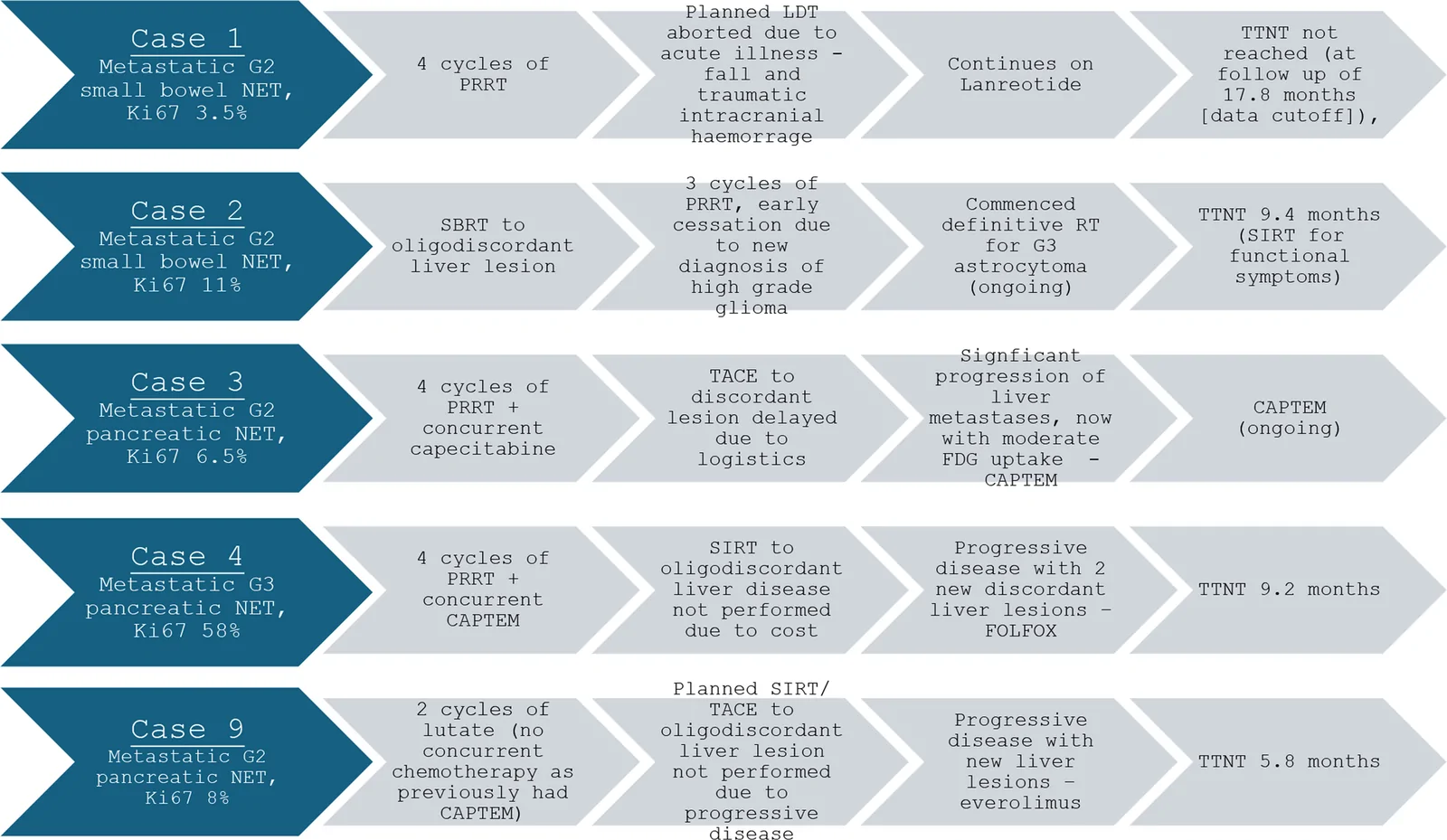
Peri-PRRT treatment sequencing in select patients who did not receive full planned treatment course

**Fig. 4:**
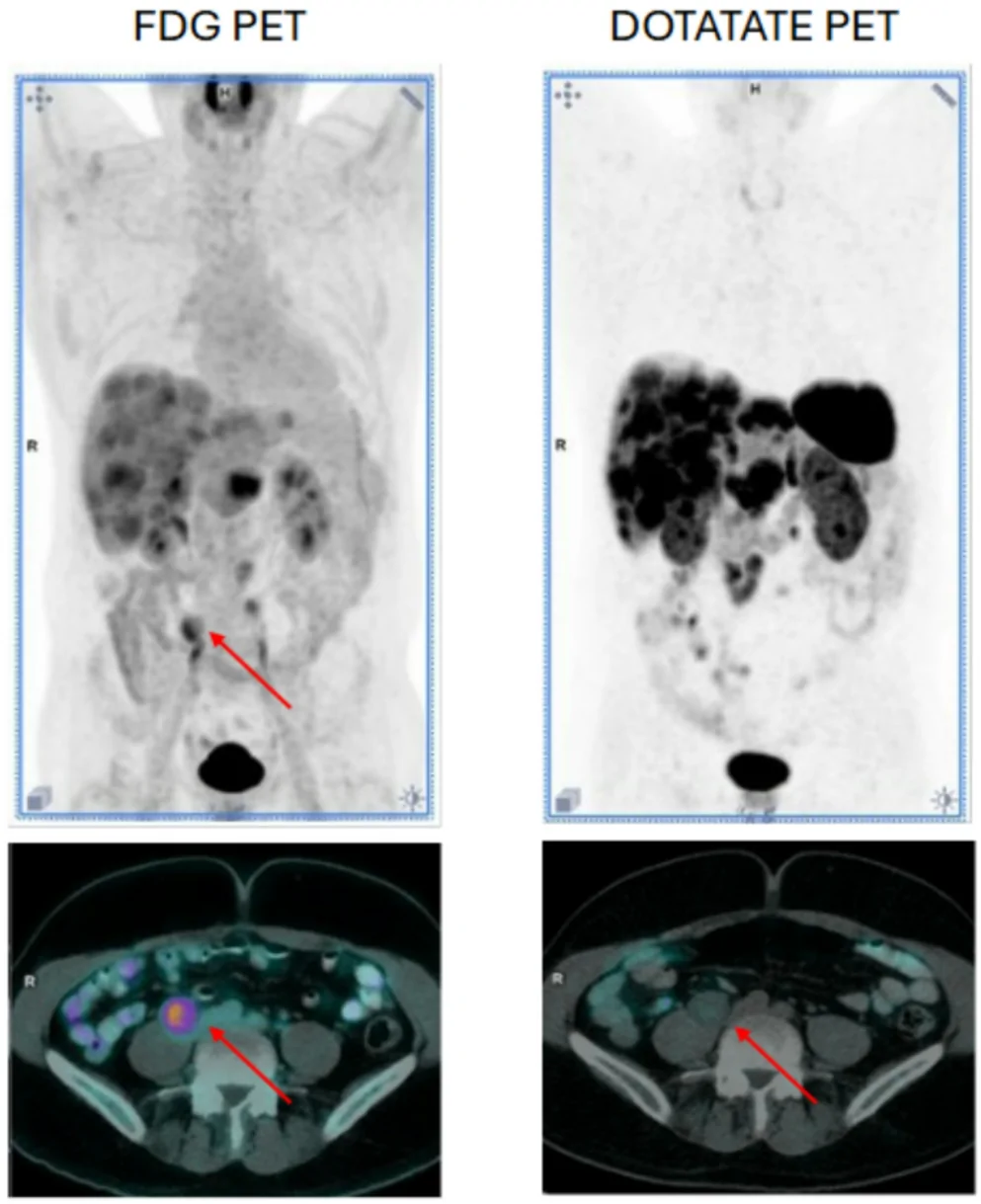
Case example. Solitary site of “oligodiscordant” disease in lymph node anterior to left psoas muscle, which displays FDG avidity but no DOTATATE avidity (red arrows). Remaining disease in small bowel primary, metastatic disease in mesentery and peritoneum show intense DOTATATE avidity (as displayed by maximum intensity projection image on top right) as well as moderate FDG avidity (top left image). The patient proceeded to receive SBRT (25Gy in 5 fractions with integrated boost to gross tumour volume to 30Gy) to the oligodiscordant lesion followed by 4 cycles of PRRT. The patient remains well 58 months later at time of data cutoff on the 9 Oct 2025

During PRRT, eight patients received concurrent chemotherapy, with the majority (6) receiving capecitabine and 2 receiving capecitabine and temozolomide. Of the 13 patients, 12 were planned for further treatment in combination with PRRT to address the oligodiscordant disease – treatment sequencing of selected cases are described in Fig. 2 and selected case example is depicted in Fig. 4. Additional treatment modalities include: liver directed therapy (LDT) such as transarterial chemoembolisation (TACE), selective internal radiation therapy (SIRT), microwave ablation (MWA), stereotactic body radiotherapy (SBRT), and intensity modulated radiation therapy (IMRT). Five of the twelve patients did not receive their planned additional treatment (liver-directed therapy). Treatment details of selected cases are depicted in Fig. 3. Two of these 5 patients received chemotherapy during PRRT. Reasons for not receiving additional treatment were: patient decision (2), intracranial haemorrhage, delay due to logistical issues, and progressive disease (1 each). Seven of the twelve patients completed these planned therapies. On completion of PRRT, 4 (31%) received LDT (TACE – 3, SIRT – 1), 3 (23%) had radiotherapy (SBRT), and 1 (8%) had both SBRT and LDT (TACE).

At the time of data cutoff, 1 patient had myelodysplastic syndrome confirmed by bone marrow biopsy, which was diagnosed approximately 6 months post the first cycle of PRRT. No other toxicities were observed beyond mild (grade 1–2), and transient cytopenia. No patients had significant grade 3 or 4 renal toxicity (Fig. 4).

**Table 2:** Treatment characteristics and outcomes

Case	Primary site	Ki-67 index, WHO Grade 2022	Treatment indication	Number of, site of oligodiscordant disease	Prior PRRT	Previous treatments (other than PRRT)	Completion of planned 4 cycles of PRRT, total dose	Reason for early cessation of PRRT	Concurrent chemotherapy with PRRT	Peri-PRRT treatment (defined as during or within 3 months before or after PRRT)	PFS (months)	OS (months), if NR (follow up in months)
1	Small bowel	3.5% G2	PD	2 Liver	Yes	SSA, SIRT, Denosumab	Yes 30.8 GBq	NA	Nil	Nil (planned for SIRT to discordant lesions; did not receive due to traumatic intracranial haemorrhage)	NR (17.8)	NR (17.8)
2	Small bowel	11% G2	PD	1 Liver	No	Surgery, SSA, TACE	No 22.8 GBq	New diagnosis of high grade glioma	Nil	SBRT to discordant liver lesion	NR (8.7)	NR (11)
3	Pancreas	6.5% G2	PD	1 Liver	No	Surgery, SSA	Yes 31.2 GBq	NA	Cape	Nil (planned TACE delayed due to logistics, later aborted due to treatment response)	11.7	NR (12)
4	Pancreas	58% G3	PD	1 Liver	No	Chemotherapy (carboplatin, etoposide)	Yes 31.7 GBq	NA	CAPTEM	Nil (SIRT suggested but prohibited by cost to patient)	8.3	NR (9.2)
5	Small bowel	11% G2	PD	1 Liver	Yes	SSA	Yes 31.5 GBq	NA	Cape	LDT (TACE) to discordant liver lesion	25.4	NR (42.3)
6	Rectal	11% G2	PD	1 Liver	Yes	SSA, TACE	No 23.6 GBq	Significant fatigue post Lutate	Nil	Nil (declined TACE)	8.6	NR (9)
7	Small bowel	37% G3	PD	1 Liver	No	Surgery, SSA, TACE, SIRT, CAPTEM	Yes 30.4 GBq	NA	Cape	LDT (TACE) to discordant liver lesion	9.6	23.4
8	Small bowel	10% G2	PD	1 Liver	No	SSA, SIRT, TACE, CAPTEM	No 15.8 GBq	PD	Cape	LDT (SIRT) to discordant liver lesion	15.1	23.4
9	Pancreas	8% G2	PD	1 Liver	No	Surgery, SSA, CAPTEM	No 15.4 GBq	PD	Nil	Nil. Planned for LDT but due to PD, PRRT was ceased after 2 cycles and everolimus commenced	4.2	9.8
10	Colorectal	5% G2	PD	1 Liver	Yes	SSA, Chemotherapy (carboplatin etoposide)	Yes 30.4 GBq	NA	CAPTEM	TACE to discordant liver lesion	4.6	23.1
11	Pancreas	12% G2	PD	1 Liver	No	SSA, SIRT, Chemotherapy (carboplatin etoposide, FOLFOX)	No 23.3 GBq	Diagnosis of myeolysplastic syndrome (MDS)	Cape	SBRT to discordant liver lesion	7.1	19.7
12	Small bowel	10% G2	PD	1 Lung	Yes	Surgery, SSA, TACE, zoledronic acid	Yes 31.0 GBq	NA	Nil	Nil	20.1	27.3
13	Small bowel	23% G3	PD	1 Lymph node	No	Surgery, SSA, SIRT	Yes 29.9 GBq	NA	Cape	SBRT to discordant nodal disease	38.1	58.6

PD – progressive disease; SSA – somatostatin analogues; LDT – liver directed therapy; SIRT – selective internal radiation therapy; TACE – trans-arterial chemo-embolization; Cape – capecitabine; CAPTEM – capecitabine and temozolomide; FOLFOX – fluorouracil and oxaliplatin; NR – not reached

### Efficacy

By data cutoff, no patients were lost to follow up, and 11 of the 13 patients had progressed, with 9 having commenced new treatment. Eleven of the patients remain alive at time of analysis. Median follow-up for patients still alive was 19.7 months.

**Fig. 5:**
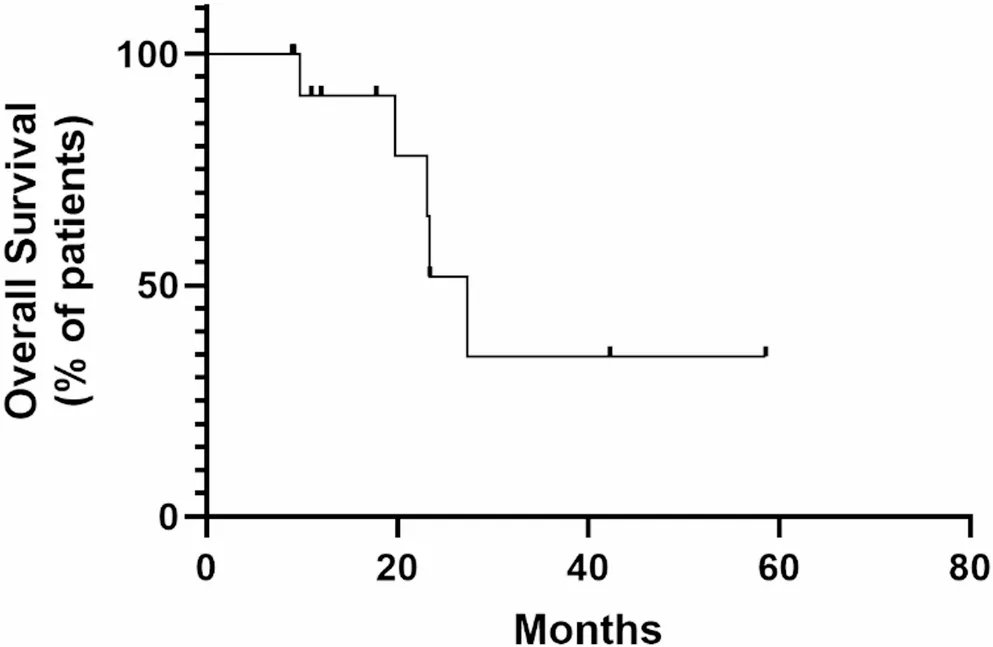
Overall survival. Kaplan-Meier survival curve overall survival of GEPNET patients with oligodiscordant metastatic disease on dual PET imaging, treated with PRRT

**Fig. 6:**
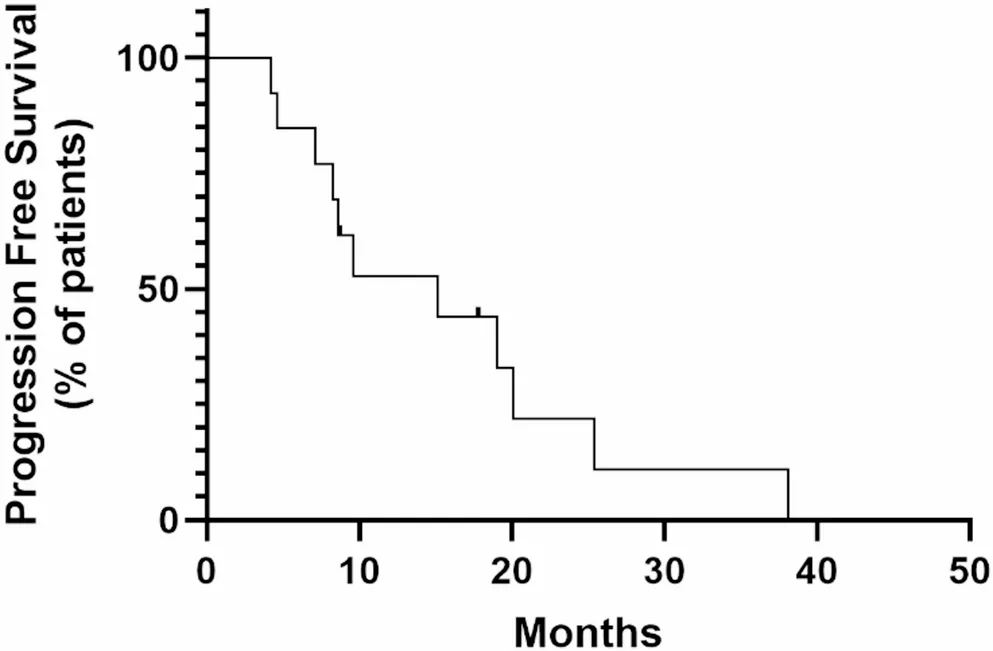
Progression free survival. Kaplan-Meier survival curve progression free survival of GEPNET patients with oligodiscordant metastatic disease on dual PET imaging, treated with PRRT

**Fig. 7:**
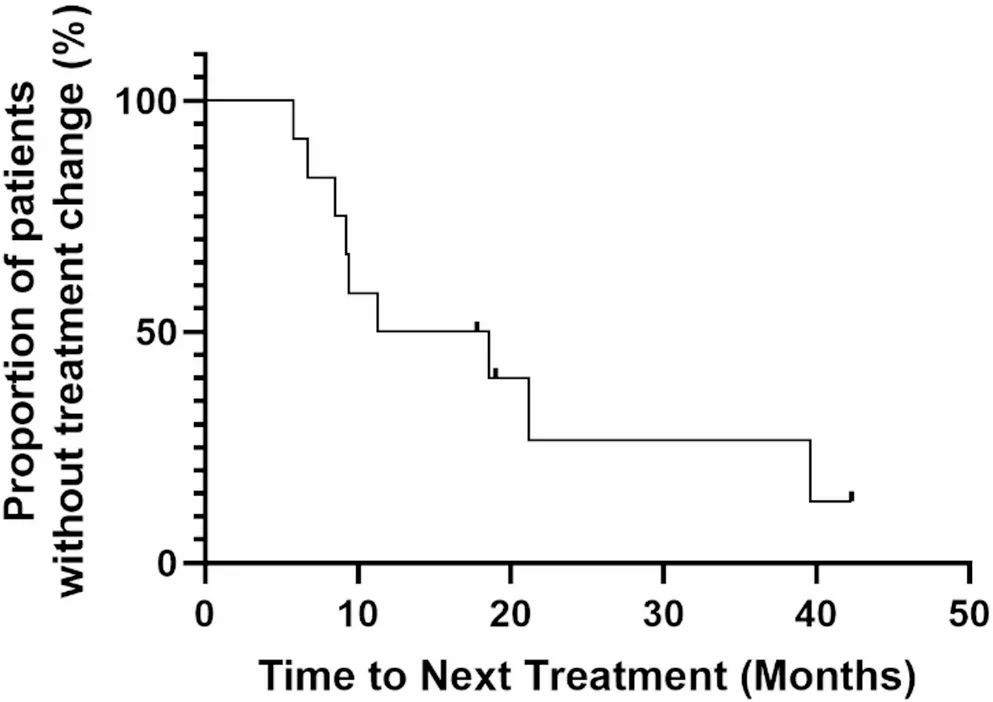
Time to next treatment. Kaplan-Meier survival curve time to next treatment of GEPNET patients with oligodiscordant metastatic disease on dual PET imaging, treated with PRRT

For the cohort, calculated mOS was 27.3 months (95% CI 19·7 - not reached) (Fig. 5), mPFS was 15.1 months (95% CI 7.1–20.1) (Fig. 6), and median TTNT was 15.0 months (95% CI 6.7–39.6) (Fig. 7).

## Discussion

We present a review of PRRT efficacy in patients with metastatic GEPNETs who have oligodiscordant disease. To our knowledge, this is the first such data to be presented for PRRT treatment in patients with lesions that are not DOTATATE avid. The role for PRRT in NETs continues to evolve and expand, with increasing evidence of its utility in more aggressive disease, including higher grade tumours (NETTER-2 trial), and those demonstrating FDG avidity [18]. The subgroup of patients with oligodiscordant disease is not represented in pivotal trials, as both NETTER-1 and NETTER-2 required all tumour lesions to be DOTATATE PET (or scintigraphy) avid for inclusion.

Whilst these patients with discordant disease are known to have worse outcomes [10, 11, 19–21] they may still potentially derive clinical benefit from PRRT administration where the majority of lesions are DOTATATE avid, alongside concomitant therapy such as chemotherapy, liver-directed therapy, radiotherapy or even surgery for the oligodiscordant lesions (Fig. 2). We therefore sought to describe outcomes from this new combined therapeutic approach.

This cohort represents a population with particularly poor prognostic features, including discordant disease, higher grade tumours, and significant pre-treatment including prior PRRT exposure. Most of the patients received concurrent chemotherapy alongside PRRT, a trend which likely reflects treatment intensification for more aggressive tumour biology, and accounts for emerging tumoral/clonal heterogeneity (with sites of more metabolically avid disease which may be less responsive to PRRT alone).

Despite expectations of poor prognosis and response to PRRT in our patient cohort, the results showed reasonable treatment outcomes. Median OS was favourable at 27.3 months, compared to 11.5 months for discordant disease in a recently published study by some of the current authors [10]. This was a multi-centre study in 2022 across a large cohort of patients who received dual PET imaging in Australia and the United Kingdom, which reported median OS by NETPET score (with P5 denoting significant discordant disease): 101.8 months for P1, 46.5 months for P2–4, and 11.5 months for P5 (*p* < 0.001) [10]. The median TTP by NETPET score was 25.5 months for P1, 16.7 months for P2–4, and 6.6 months for P5 (*p* < 0.001). TTNT was marginally shorter than PFS, which likely reflects the treatment changes that were driven by other clinical factors rather than disease progression (such as ongoing symptoms necessitating further treatment). Thus, our experience supports the safety and efficacy of PRRT for patients with oligodiscordant disease. While this study primarily reported on efficacy outcomes, there may also be important patient-centered benefits such as maintaining quality of life and tolerability of treatment, given the overall favourable side effect profile of PRRT.

The recently published CABINET trial, which included a similar patient group (higher grade disease, and a heavily pre-treated population), was associated with a shorter median PFS of 8.4 months (extra-pancreatic group) and 13.8 months (pancreatic group) [22]. Whilst the PFS and OS in NETTER-1 and NETTER-2 are numerically higher than the results from our small series, our cohort differs in that patients had higher grade tumours than the patients in NETTER-1 and had received multiple lines of prior treatment than the patients in NETTER-2.

Our study illustrates how dual PET imaging can guide management in patients with NETs. Whilst histological tumour grade, including mitotic rate and the Ki*-*67 index are important clinical parameters, the presence of discordant disease also portends higher aggressiveness and a lack of a target in the discordant lesion for PRRT. FDG PET is particularly important in higher grade tumours, current literature shows that up to 40% of patients with G1 disease also have FDG avidity [18, 21]. The lower number of patients with G3 disease in our cohort may reflect the fact that patients with G3 disease may have many more discordant lesions (and thus be truly unsuitable for PRRT). Although dual PET imaging does utilize scarce health care resources, the savings from avoiding futile PRRT in patients with multiple discordant lesions may offset this cost.

This study also provides some early insight into the feasibility of combining different types of treatment for patients who display tumour heterogeneity in a multidisciplinary setting [23]. As an accredited ENETs Centre of Excellence, we routinely review all patients with a GEPNEN diagnosis, and mandate dual PET imaging prior to final determination regarding PRRT suitability. The varied treatment modalities employed alongside PRRT in our cohort emphasises the fact that there are no specific guidelines, or consensus recommendations for treatment combination/sequencing in this patient group. Treatment approach is chosen on an individual basis in an MDT setting after considering multiple factors, including site of oligometastatic disease, overall burden of disease, cadence of progression, logistics and patient fitness.

The strengths of this study include its description of real-world practice at a tertiary referral ENETS Centre of Excellence with standardised imaging protocols, treatment delivery and follow-up imaging. It provides a novel treatment algorithm in this rare subgroup of patients with limited treatment options. The cohort included rural and regional patients at our site, and no patients were lost to follow-up. We acknowledge the several limitations of our study – its retrospective design, small sample size and lack of a control group, all of which limits the ability to draw definitive conclusions regarding treatment benefit. A larger sample size, and comparison with a propensity matched cohort would strengthen assessment of treatment outcomes and may permit subgroup analyses, including the assessment of the optimal lesion number to define oligodiscordant disease. Lastly, this study captured patients receiving treatment over a period of significant disruption to the health system during the COVID-19 pandemic from 2020 to 2023, which may have led to potential disruptions and/or delays in diagnosis, imaging and delivery of planned treatments. We note that there was 1 patient who did not receive planned LDT (TACE) due to multiple treatment delays in the COVID-19 pandemic era.

Providing a new treatment option for NET patients with “oligodiscordant” disease is especially important in Australia as treatment options beyond PRRT are limited, with the Pharmaceutical Benefits Scheme subsidy for everolimus being limited to those with a pancreatic primary and cabozantinib only becoming available recently [24, 25]. Given the higher grade of tumours as well as poorer prognosis conferred by discordant disease, these patients often receive chemotherapy, but this can be poorly tolerated, especially compared to the more favourable side effect profile of PRRT.

A prospective study, preferably multi-centre with longer follow up, would be ideal to validate our findings, though accrual may be difficult given small patient numbers and funding for adequate follow-up may be scarce. Future studies could also incorporate radiomics for additional information which may be useful for prognostication and patient selection, such as volume of oligodiscordant disease, and its ratio compared to volume of DOTATATE avid disease, and SUV_max_ of FDG avid disease [26].

## Conclusion

PRRT is a highly effective and tolerable treatment option in patients with advanced GEPNETs. We present data showing that PRRT can be effective with an acceptable safety profile in selected patients with oligodiscordant disease found on dual PET imaging (FDG and DOTATATE). Given these patients would not have been eligible for inclusion in the NETTER-1/NETTER-2 studies, there is a lack of current evidence-informed treatment guidelines for this subset of patients. Whilst awaiting further, we suggest that PRRT could be considered in carefully selected patients with oligodiscordant disease, where treatment exists to address the oligodiscordant disease, when discussed by a multidisciplinary team in a centre experienced in treating NETS. Treatment decisions and sequencing in this complex situation should be made on an individualised basis. Multi-modality treatment with PRRT in combination with local therapies (LDT, radiotherapy or surgery) or chemotherapy may be considered as an emerging treatment paradigm in oligodiscordant NET.

## Data Availability

The datasets generated and analysed for the current study are available from the corresponding author on reasonable request.
